# *Arenaria serpyllifolia* as a Natural Antiviolaceum Agent: Phytochemical, Biological, and Molecular Approaches

**DOI:** 10.1002/open.202500236

**Published:** 2025-07-15

**Authors:** Meryem Burcu Külahcı, Betül Aydın, Emine Incilay Torunoğlu, Zekeriya Düzgün, Alper Durmaz, Erdi Can Aytar

**Affiliations:** ^1^ Faculty of Science Department of Biology Gazi University 06500 Ankara Türkiye; ^2^ Faculty of Medicine Department of Medical Biochemistry Necmettin Erbakan University 42090 Konya Türkiye; ^3^ Faculty of Medicine Department of Medical Biology Giresun University 28100 Giresun Türkiye; ^4^ Ali Nihat Gökyiğit Botanical Garden Application and Research Center Artvin Çoruh University 08000 Artvin Türkiye; ^5^ Faculty of Agriculture Department of Horticulture Usak University 64200 Uşak Türkiye

**Keywords:** antibiofilm activity, antimicrobial activity, antioxidant activity, antiquorum sensing, *Arenaria serpyllifolia*, molecular docking, molecular dynamics simulations

## Abstract

This study investigates the antioxidant, antimicrobial, antibiofilm, and antiquorum sensing activities of *Arenaria serpyllifolia* extract. A methanolic extract from the plant's above‐ground parts is prepared via maceration. The extract exhibits strong antioxidant properties (DPPH IC_50_: 355.54 ± 20.62 μg mL^−1^) and iron chelating ability (IC_50_: 5.30 ± 4.44 mg mL^−1^). Total flavonoid and phenolic contents are 75.15 ± 2.73 mg quercetin equivalent g^−1^ and 150.83 ± 11.24 mg gallic acid equivalent g^−1^, respectively. Antimicrobial tests show notable activity against *Chromobacterium violaceum* (minimal inhibitory concentration (MIC) < 5 mg mL^−1^). Antibiofilm effects are significant with 82.52% and 81.32% inhibition at MIC and sub‐MIC levels. The extract also inhibits violacein production in the *C. violaceum* CV12472 strain (90.76% at MIC). Gas chromatography‐mass spectrometry analysis identifies seven major compounds, including allyl isothiocyanate and levoglucosan. Molecular docking reveals levoglucosan as the most potent CviR receptor binder (−6.8 kcal mol^−1^). These interactions suggest possible quorum sensing inhibition via antagonism. Molecular dynamics simulations confirm the stability of the ligand–receptor complexes, highlighting guanosine and levoglucosan as promising leads for drug development.

## Graphical abstract

*Arenaria serpyllifolia* extract exhibits strong antioxidant, antimicrobial, antibiofilm, and antiquorum sensing activities. Key compounds, including levoglucosan and guanosine, show potent CviR receptor binding. Violacein inhibition and molecular dynamics confirm their stability, highlighting their potential as quorum sensing inhibitors and therapeutic leads.

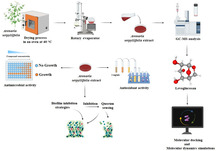

## 1 Introduction

Throughout history, humans have depended on plants for various purposes, including food, medicine, woodworking, fertilizer, and aromatic substances. The increasing use of natural compounds encourages the development of suitable extraction methods. Additionally, significant advancements have been made in characterizing valuable phytochemicals in plant extracts, contributing to ecological sustainability and the discovery of safer therapeutic agents.^[^ ^1^ ^]^ Plant extracts are rich in secondary metabolites such as flavonoids, alkaloids, terpenoids, and tannins, exhibiting diverse biological activities that make them valuable in traditional medicine.^[^ ^2^ ^]^ However, despite their increasing use for medicinal purposes, many plant extracts and their chemical components remain unidentified, with some evaluated only for their biological activities.^[^ ^3^ ^]^ Due to their various health benefits, medicinal plants and natural products have become a focal point of modern scientific research. Some have been approved as safe chemotherapy drugs, while others have been recognized as natural antioxidants, prompting researchers to seek new, nontoxic alternatives to replace harmful synthetic compounds.^[^ ^4^ ^]^

*Arenaria serpyllifolia* L., in *Sp. Pl.*: 423 (1753) is an annual herbaceous plant belonging to the family Caryophyllaceae. The genus *Arenaria* comprises 188 taxonomically accepted species worldwide, and *A. serpyllifolia* is classified as one of these species. The genus name *Arenaria* is derived from Latin, meaning “growing in sandy places,” while the specific epithet *serpyllifolia* refers to the resemblance of the leaves to those of thyme (*Thymus serpyllum*). Two homotypic synonyms of the species are listed in taxonomic sources: *Alsinanthus serpyllifolius* (L.) Desv. and *Stellaria serpyllifolia* (L.) Scop., indicating that the plant was previously placed in different genera. However, today the valid scientific name *A. serpyllifolia* L. is accepted based on Linnaeus's original description.^[^ ^5^, ^6^, ^7^ ^]^

Antimicrobial resistance (AMR) is a major issue for human health. Bacteria, viruses, fungi, and parasites change over time and no longer respond to medications. This makes infections harder to treat and increases the risk of disease spread. AMR leads to higher mortality rates in hospitals and requires more expensive medications and longer hospital stays, which in turn raise healthcare costs. In this context, natural antimicrobial compounds derived from plants are considered a potential alternative in the fight against AMR.^[^ ^8^ ^]^ Pharmaceutical products and their derivatives have been found in the environment, water bodies, and treated waters. Azithromycin (AZT) is a broad‐spectrum antibiotic. It is widely used against respiratory pathogens. This drug is mostly excreted from the body unchanged. The presence of AZT in the environment causes negative impacts on the ecosystem and human health.^[^ ^9^ ^]^ Plant extracts may have antibacterial and antifungal properties, which can help develop new and effective approaches to infection treatment. Thus, plant‐based resources may offer safer and more effective treatment options against resistant pathogens.

Violacein is a naturally occurring bisindole‐derived pigment that exhibits a violet coloration. Since its discovery, violacein‐producing microorganisms have been screened and identified. Among these, *Chromobacterium violaceum* is a model organism for violacein production.^[^ ^10^ ^]^ Microbial pigment production acts as a protective defense mechanism against environmental threats. Bacteria produce pigments and antibiotics to kill invading bacteria and protect their resources. These pigments help preserve microbial species in environments, like soil and water. Biopigments are beneficial natural molecules that offer protective and resistant mechanisms against toxic bacteria, cancer cells, parasites, and biological threats.^[^ ^11^ ^]^ Quorum sensing (QS) plays a crucial role in regulating violacein production, as the QS mechanism coordinates the pigment production in violacein‐producing microorganisms like *C. violaceum* and provides protective defense against environmental conditions.^[^ ^12^ ^]^

QS is a cellular communication mechanism used to coordinate collective behaviors among microorganisms. In this process, bacteria and other organisms communicate through small molecules called quorum sensing molecules (QSM), also known as autoinducers, which are released into the environment and accumulate to a certain density. QS regulates various biological processes such as biofilm formation, exoenzyme secretion, antimicrobial substance synthesis, and the expression of virulence factors. Microorganisms establish complex interaction networks in natural or artificial ecosystems. These interactions primarily involve cooperation and competition relationships, directly influencing and driving the microbial community. QS determines microbial interaction modes by regulating behaviors, such as cross‐feeding and the secretion of antimicrobial substances.^[^ ^13^ ^]^

The CviR receptor is a protein involved in the QS system of the bacterium *C. violaceum*. This system allows bacteria to regulate gene expression based on their population density. The CviR receptor is part of a regulatory system that controls processes such as the production of violacein, a purple pigment produced by *C. violaceum*. In *C. violaceum*, CviR acts as a response regulator that binds to autoinducer molecules produced by the bacteria.^[^ ^14^ ^]^ This binding initiates the transcription of genes that control various processes, such as biofilm formation, virulence (disease‐causing potential), and pigment production. Interference with the interaction between the CviR receptor and the autoinducers can inhibit QS, thereby reducing bacterial virulence and biofilm formation. Therefore, the CviR receptor may serve as a potential target for antimicrobial agents developed to control bacterial infections. When testing quorum sensing inhibitor (QSI) compounds, it is crucial to perform the minimal inhibitory concentration (MIC) tests accurately, as at higher concentrations, the compound may exhibit antibacterial effects. In QS inhibition studies, in silico analyses and tests using biosensor strains provide interesting findings with these compounds.^[^ ^15^ ^]^

This study aims to determine the antioxidant, antimicrobial, antibiofilm, and anti‐QS activities of *A. serpyllifolia* extract, identify its chemical composition through gas chromatography‐mass spectrometry (GC‐MS) analysis, evaluate the interactions of its components with the CviR receptor via molecular docking studies, and analyze the binding stability of ligand–target complexes using molecular dynamics (MD) simulations.

## 2 Results and Discussion

### 2.1 Antioxidant Activity, Iron Chelating Properties, and Flavonoid Content of *A. serpyllifolia* Extract

The IC_50_ value of the DPPH radical scavenging activity of *A. serpyllifolia* extract was determined to be 355.54 ± 20.62 μg mL^−1^, and the IC_50_ value for iron chelation activity was 5.30 ± 4.44 mg mL^−1^. These results indicate that the extract exhibits good antioxidant activity and strong iron chelation activity. Additionally, the total flavonoid content of *A. serpyllifolia* extract was measured to be 75.15 ± 2.73 mg quercetin equivalent (QE) g^−1^ extract, total phenolic content of extract was measured to be 150.83 ± 11.24 mg gallic acid equivalent (GAE) g^−1^ extract demonstrating that the extract has a significant flavonoid content (**Table** **1**).

**Table 1:** Antioxidant and iron chelating activities, total flavonoid of *A. serpyllifolia* extract (mean ± SD; *n* = 3).

	DPPH Assay (IC_50_; [μg mL^−1^])	Iron chelating activity (IC_50_; [mg mL^−1^])	Total flavonoid content [mg QE g^−1^ extract]	Total phenolic content [mg GAE g^−1^ extract]
*Arenaria serpyllifolia*	355.54 ± 20.62	2.01 ± 0.36	75.15 ± 2.73	150.83 ± 11.24
BHT	230 ± 10	–	–	–
EDTA	–	5.30 ± 4.44	–	

Oxidative stress can result from an imbalance between antioxidants and free radicals in the body,^[^ ^16^ ^]^ and plants contain antioxidants that may play a potential role in preventing oxidative damage.^[^ ^17^ ^]^ The antioxidant effect of *A. serpyllifolia* extracts was evaluated using the DPPH and iron chelation assays. The DPPH assay yielded a value of 355.54 ± 20.62 μg mL^−1^, indicating that the methanol extract exhibits good antioxidant activity. The iron chelation activity was determined to be 2.01 ± 0.36 mg mL^−1^, demonstrating a strong effect. The iron chelation scavenging effect has the potential to reduce oxidative stress by binding free iron ions.^[^ ^18^ ^]^ The DPPH and iron chelation activities may be attributed to the presence of specific chemical compounds such as glycerin,^[^ ^19^ ^]^ allyl isothiocyanate,^[^ ^20^ ^]^ guanosine,^[^ ^21^ ^]^ and levoglucosan,^[^ ^22^ ^]^ which exhibit free radical activity. The chemical compounds found in *A. serpyllifolia* extracts suggest that they may act as free radical scavengers and metal ion chelators, contributing to their antioxidant properties. Natural antioxidants are considered safer compared to synthetic ones.^[^ ^23^ ^]^ Variations in antioxidant activity have been observed among *Arenaria* plant extracts. This difference may be attributed to the chemical compounds present in the plant. These compounds can exhibit synergistic effects, leading to different antioxidant behaviors. Variability in chemical composition may influence antioxidant activity. Antioxidant studies have been conducted on *A. serpyllifolia* extracts; however, research on the *Arenaria* genus remains limited. In the study conducted by Kazemi and Firouzeh (2022),^[^ ^24^ ^]^ the IC_50_ value for the antioxidant activity of *Arenaria hispanica* extract was determined to be 0.69 ± 0.03 mg mL^−1^, whereas in our study, the antioxidant activity of *A. serpyllifolia* extract was reported to be IC_50_ value 355.54 ± 20.62 μg mL^−1^, indicating a higher antioxidant activity of *A. serpyllifolia.* The IC_50_ value of the DPPH radical scavenging activity of *Arenaria montana* methanol extract was determined to be 0.90 ± 0.01 mg mL^−1^, as reported by Pereira et al. (2014),^[^ ^25^ ^]^ while the antioxidant activity of *A. serpyllifolia* extract was found to be 355.54 ± 20.62 μg mL^−1^. This result indicates that *A. serpyllifolia* exhibits higher antioxidant activity. The differences in antioxidant activity can be attributed to environmental factors, the plant species, climatic conditions, the use of different plant parts, extraction methods, and the applied extraction conditions.^[^ ^4^ ^]^

### 2.2 Antimicrobial, Antibiofilm, and QS Activity of *A. serpyllifolia* Extract

The MIC, minimum bactericidal concentration (MBC), and minimum fungicidal concentration (MFC) values of *A. serpyllifolia* extract against bacterial and fungal isolates are presented in **Table** **2**. The MIC value of the extract against *C. violaceum* bacterial isolates was found to be <5 mg mL^−1^. This indicates that the extract exhibits good antimicrobial activity against the *C. violaceum* bacterial isolate.

**Table 2:** MIC, MBC, and MFC values of *A. serpyllifolia* extract.

Organism	MIC [mg mL^−1^]	MBC/MFC [mg mL^−1^]	MIC/MBC/MFC [μg mL^−1^]	PC [μg mL^−1^]a)
*Staphylococcus aureus* ATCC 25923	12.5	<50	1	64
*Bacillus cereus* NRRL B‐3711	<25	25	4	>8
*Escherichia coli* ATCC 25922	<50	50	1	>1
*Pseudomonas aeruginosa* ATCC 27853	<50	50	64	>64
*Pseudomonas aeruginosa* PA01	<50	50	64	>64
*Chromobacteriumviolaceum* ATCC 12472	<5	5	4	>4
*Candida albicans* ATCC 10231	50	>50	2	>2

a) AZT for bacteria, ketoconazole for yeast strain.

*A. serpyllifolia* extract inhibited biofilm formation by 82.52 ± 2.2% at the MIC dose of 5 mg mL^−1^. The extract showed the best effect at the sub‐MIC dose of 4 mg mL^−1^, inhibiting biofilm formation by 81.32 ± 2.4%. These results indicate that *A. serpyllifolia* extract exhibits strong antimicrobial biofilm activity (**Table** **3**).

**Table 3:** Concentration‐dependent inhibition percentage of *A. serpyllifolia* extract on biofilm formation.

	Concentration	*P. aeruginosa* PAO1	Concentration	*C. violaceum* ATCC 12472
MIC dose	50 [mg mL^−1^]	72.77 ± 2.8a)	5 [mg mL^−1^]	82.52 ± 2.2
Sub‐MIC doses	40 [mg mL^−1^]	62.42 ± 2.3	4 [mg mL^−1^]	81.32 ± 2.4
	30 [mg mL^−1^]	51.30 ± 1.1	3 [mg mL^−1^]	80.27 ± 3.2
	20 [mg mL^−1^]	53.57 ± 2.3	2 [mg mL^−1^]	52.73 ± 1.7
	10 [mg mL^−1^]	–	1 [mg mL^−1^]	42.85 ± 2.3
MIC dose of PCb)	64 [μg mL^−1^]	90.27 ± 1.9	4 [μg mL^−1^]	89.83 ± 3.5

a) (mean ± SD).

b) (Positive control, AZT; PC).

**Figure 1:**
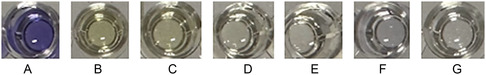
Violacein inhibition on *C. violaceum* ATCC 12472 by *A. serpyllifolia* extract. * A) Negative control, B) 5 mg mL^−1^, C) 4 mg mL^−1^, D) 3 mg mL^−1^, E) 2 mg mL^−1^, F) 1 mg mL^−1^, and G) positive control (AZT).

The potential QS inhibition of plant extracts was evaluated using *C. violaceum* CV12472 bacteria, which produce purple violacein during growth (**Table** **4**). The MIC, defined as the lowest concentration at which visible bacterial growth was inhibited by the extracts, was determined, and violacein inhibition and QS assays were performed at MIC and sub‐MIC levels. The MIC value for the methanol extract against *C. violaceum* CV12472 was determined to be 5 mg mL^−1^. At the MIC level, the ethanol extracts inhibited violacein production by 90.76 ± 0.3%. At sub‐MIC levels, the violacein production inhibition percentages of the methanol extract were determined as 91.60 ± 0.1%, 91.25 ± 0.0%, 90.83 ± 0.6%, and 80.88 ± 1.9%, respectively (**Figure** **1**).

**Table 4:** Violacein production inhibition percentage of *A. serpyllifolia* extract on *C. violaceum* ATCC 12472.

	Sub‐MIC Doses					
	5 [mg mL^−1^]	4 [mg mL^−1^]	3 [mg mL^−1^]	2 [mg mL^−1^]	1 [mg mL^−1^]	PCa)
*Arenaria serpyllifolia* extract	90.76 ± 0.3	91.60 ± 0.1	91.25 ± 0.0	90.83 ± 0.6	80.88 ± 1.9	93.72 ± 0.3

a) PC: AZT (MIC dose; 4 [μg mL^−1^]).

The effect of *A. serpyllifolia* extracts on QS‐mediated processes, such as the inhibition of violacein production in *C. violaceum* CV12472, has been investigated. This plant not only kills bacteria but can also reduce their severity by disrupting QS networks. Additionally, it may eliminate bacterial resistance. Antibiotics based solely on bacterial growth inhibition or bactericidal effects can lead to health problems due to the development of resistant strains.^[^ ^26^, ^27^, ^28^ ^]^ Since the methanol extract inhibited violacein formation and QS, this activity can be attributed to some of the identified chemical compounds, such as glycerin,^[^ ^29^ ^]^ allyl isothiocyanate,^[^ ^30^ ^]^ guanosine,^[^ ^31^ ^]^ levoglucosan,^[^ ^32^ ^]^ and 2,2‐dimethoxybutane;^[^ ^33^ ^]^ these compounds are supported by our in silico models. *A. serpyllifolia's* antimicrobial activity, antibiofilm activity, and anti‐QS effects have been reported for the first time here. The extract of *A. serpyllifolia* shows strong antimicrobial activity (MIC, 5 mg mL^−1^) against *C. violaceum,* compared to other bacterial isolates. Therefore, *A. serpyllifolia* extracts can be used as a potential solution to reduce microbial resistance and the severity of infections.^[^ ^34^ ^]^ The extract inhibited violacein production in *C. violaceum* (sub‐MIC dose; 1 mg mL^−1^; 80.88 ± 1.9). During bacterial communication, signal molecules can diffuse within colonies, which can regulate microorganism motility. Swarming motility plays a role in QS‐mediated biofilm formation.^[^ ^26^, ^35^ ^]^ When microorganisms face challenges, such as antibiotics, they form biofilms to survive. The *A. serpyllifolia* extract inhibited biofilm activity (sub‐MIC dose; 4 mg mL^−1^), showing good antibiofilm activity against *C. violaceum* bacterial isolates. *Clinopodium nepeta* dichloromethane (DCM) extract inhibited violacein production in *C. violaceum* CV12472 at a MIC concentration of 0.5 mg mL^−1^ (100.0 ± 0.0%), while the ethyl acetate (AcOEt) extract exhibited inhibition at a MIC concentration of 1 mg mL^−1^ (18.9 ± 4.1%). At MIC/2, the percentage inhibition of violacein production was 19.2 ± 1.1% and 4.5 ± 0.3% for DCM and AcOEt extracts, respectively.^[^ ^4^ ^]^ In our study, the MIC value of *A. serpyllifolia* extract was determined as 5 mg mL^−1^, showing an inhibition of 90.76 ± 0.3%. Dose‐dependent inhibition rates at sub‐MIC concentrations (1–4 mg mL^−1^) were recorded as 91.60 ± 0.1% and 80.88 ± 1.9%, respectively. Both studies demonstrated the ability to disrupt bacterial QS. In our study, *A. serpyllifolia* extracts, like the extracts of *Searsia leptodictya, Searsia lancea, Searsia batophylla, Searsia pendulina, Bauhinia galpinii*, and *Bauhinia bowkeri*, exhibited antibacterial and antibiofilm activity and were found to modulate the QS mechanism by inhibiting violacein production.^[^ ^36^ ^]^

### 2.3 Chemical Profiling of *A. serpyllifolia* via GC‐MS Analysis

GC‐MS analysis of the methanolic extract of *A. serpyllifolia* revealed the presence of several semipolar phytochemical constituents (**Table** **5**). A total of seven major compounds were identified, each eluting at distinct retention times. The predominant compound was allyl isothiocyanate, accounting for 23.59% of the total peak area, followed by 2,2‐dimethoxybutane at 22.28%. Notable quantities of levoglucosan (18.71%) and methyl butyrate (12.94%) were also detected. Additionally, guanosine (7.58%), glycerin (6.75%), and 2,6,7‐trimethyldecane (3.59%) were present in relatively lower proportions. These findings suggest that the methanolic extract of *A. serpyllifolia* contains a diverse array of semipolar compounds, reflecting its rich phytochemical profile.

**Table 5:** GC‐MS analysis results of *A. serpyllifolia* methanol extract.

No	Retention time [min]	Compound name	Area [%]
1	3.244	Methyl butyrate	12.94
2	3.451	Glycerin	6.75
3	3.890	2,2‐dimethoxybutane	22.28
4	7.404	Allyl isothiocyanate	23.59
5	14.785	2,6,7‐trimethyldecane	3.59
6	24.466	Guanosine	7.58
7	25.841	Levoglucosan	18.71

Although studies have been conducted on *A. serpyllifolia* extracts, the chemical composition of this plant has not yet been fully elucidated. The results of the phenolic chemical composition here show that *A. serpyllifolia* methanol extracts contain various chemical compounds. Using GC‐MS analysis, seven chemical compounds were identified, and their quantities were determined.^[^ ^37^ ^]^ Careful selection of extraction methods and solvents ensures the maximum yield of target compounds found in plants and helps avoid chemical or biological alterations. In this study, methyl butyrate, glycerin, 2,2‐dimethoxybutane, allyl isothiocyanate, 2,6,7‐trimethyldecane, guanosine, or levoglucosan were detected in *A. serpyllifolia* samples collected from Turkey. In one study, 10 compounds were isolated from the extract of *Arenaria kansuensi*s.^[^ ^38^ ^]^ The chemical composition of *Arenaria montana* was found to consist of hydrophilic (sugars, organic acids, and phenolic compounds) and lipophilic (fatty acids and tocopherols) components.^[^ ^25^ ^]^ The chemical components of *A. kansuensis* were identified using high‐performance liquid chromatography (HPLC) analysis.^[^ ^39^ ^]^ Various chemical compounds of the *Arenaria* genus have been identified using different analytical methods. The compounds identified by GC‐MS in *A. serpyllifolia* represent an important study in this field.

### 2.4 Binding Affinity‐Based Evaluation of Ligand–target Interactions

**Table** **6** presents the calculated binding interaction parameters of the selected compounds with the target protein 3QP1. The table includes binding energy values (in kcal mol^−1^), ligand efficiency (LE), fit quality (FQ), estimated inhibition constants (Ki, in μM), and pIC_50_ values for each compound .

**Table 6:** Results of binding interactions of the compounds with target 3QP1.

	Binding energy [kcal mol^−1^]	Ligand efficiency	Fit quality (FQ)	Estimated inhibition constant {(Ki) [μM]}	pIC_50_
Methyl butyrate	−4.6	0.657	0.241	422.80	3.290
Glycerin	−4.2	0.700	0.188	830.83	3.000
2,2‐dimethoxybutane	−4.1	0.513	0.242	983.69	2.930
Allyl isothiocyanate	−5.1	0.850	0.229	181.72	3.640
2,6,7‐trimethyldecane	−4.7	0.362	0.374	357.10	3.360
Guanosine	−6.2	0.413	0.520	28.350	4.430
Levoglucosan	−6.8	0.618	0.498	10.293	4.860

**Figure 2:**
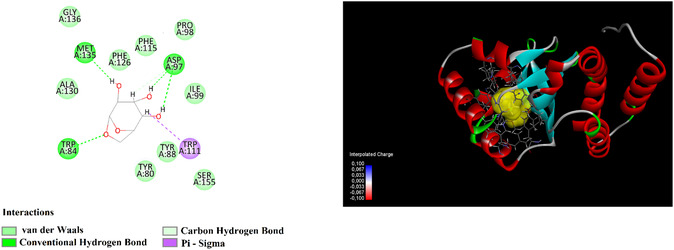
2D and 3D molecular docking interactions of levoglucosan with the target 3QP1.

Among the tested molecules, levoglucosan demonstrated the strongest binding affinity with a binding energy of –6.8 kcal mol^−1^, an estimated Ki of 10.29 μM, and a pIC_50_ value of 4.86 (**Figure** **2**). Similarly, guanosine exhibited notable interaction with the target, showing a binding energy of –6.2 kcal mol^−1^, Ki of 28.35 μM, and a pIC_50_ of 4.43, indicating favorable inhibitory potential.

In contrast, compounds such as 2,2‐dimethoxybutane, glycerin, and methyl butyrate showed significantly weaker binding affinities, with high Ki values of 983.69, 830.83, and 422.80 μM, respectively. These findings suggest that these small and more polar molecules are less effective in interacting with the active site of 3QP1.

In terms of LE, allyl isothiocyanate exhibited the highest LE value (0.850), suggesting that, despite its smaller molecular size, it binds relatively efficiently to the target site. However, its FQ was relatively low (0.229), indicating potential room for structural optimization.

Overall, levoglucosan and guanosine emerged as the most promising candidates due to their strong binding affinities and favorable efficiency metrics, suggesting their potential for further investigation in drug design targeting 3QP1.

### 2.5 Evaluation of Binding Stability through MD Simulations

To investigate the structural stability and binding characteristics of various small molecules to the ligand‐binding domain (LBD) of CviR (PDB ID: 3QP1), MD simulations spanning 100 ns were performed for the protein complexed with seven distinct ligands: 2,2‐dimethoxybutane, allyl isothiocyanate, levoglucosan, methyl butyrate, guanosine, glycerin, and 2,6,7‐trimethyldecane. The dynamic behavior and interaction energies were analyzed to assess system stability and predict binding affinities.

**Figure 3:**
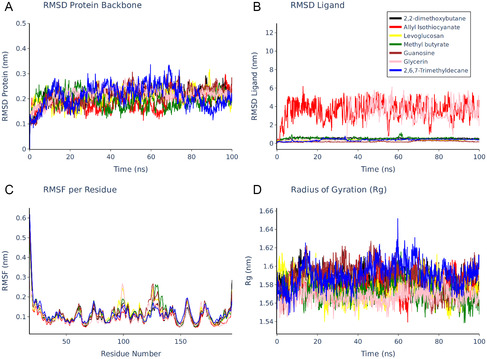
MD simulations of seven different compounds showing protein backbone stability, ligand mobility, residue fluctuations, and protein compactness over 100 ns.

Evaluation of the protein's structural integrity commenced with the analysis of the root mean square deviation (RMSD) of the backbone Cα atoms relative to the initial minimized structure (**Figure** **3**A). Across all simulated systems, the protein backbone RMSD values demonstrated convergence after an initial equilibration phase of ≈10–20 ns. Subsequently, the RMSD values remained stable, generally fluctuating within a range of 0.15–0.25 nm, with transient peaks occasionally reaching ≈0.3 nm. This indicates that the overall fold of the CviR LBD was well‐maintained throughout the 100 ns simulation period in the presence of all tested ligands, without significant conformational rearrangements. Further supporting global structural stability, the radius of gyration (*R*_g_) of the protein was monitored over time (Figure 3D). The *R*_g_ values for all systems fluctuated around a stable average, predominantly within the 1.54–1.64 nm range, confirming that the protein maintained its overall compactness and did not undergo significant unfolding or expansion events upon ligand binding.

The stability of the ligands within the CviR binding pocket was assessed by calculating the RMSD of the ligand heavy atoms after aligning the protein backbone (Figure 3B). This analysis revealed marked differences in ligand behavior. Guanosine (brown), 2,6,7‐trimethyldecane (blue), 2,2‐dimethoxybutane (black), levoglucosan (yellow), and methyl butyrate (green) exhibited relatively stable binding poses, with RMSD values generally stabilizing below 1.0 nm after initial adjustments. This suggests that these ligands maintained consistent interactions within the binding site. In striking contrast, allyl isothiocyanate (red) displayed exceptionally high and erratic RMSD values, frequently fluctuating between 2 and 6 nm and exhibiting peaks beyond this range. This pronounced instability indicates that allyl isothiocyanate failed to establish a stable binding mode and likely diffused away from, or moved extensively within, the putative binding site during the simulation. Glycerin (pink) also showed considerable mobility, with RMSD values fluctuating significantly, often exceeding 1 nm and reaching peaks near 4 nm, suggesting a less stable or transient interaction compared to the more stably bound ligands.

To probe the local dynamics and flexibility of the protein structure, the root mean square fluctuation (RMSF) was calculated for each residue over the simulation trajectory (Figure 3C). The RMSF profiles were broadly similar across all complexes, indicating that the binding of these diverse ligands did not induce major alterations in the protein's intrinsic flexibility pattern. As anticipated, regions corresponding to loops (e.g., residues ≈40–50, ≈90–110, ≈120–140, and ≈150–160) and the N‐ and C‐termini exhibited higher RMSF values, signifying greater conformational freedom. Conversely, residues located within secondary structure elements displayed lower RMSF values, reflecting their comparative rigidity. While minor variations in peak heights existed between different complexes, no ligand caused a distinct, widespread change in the flexibility profile, suggesting interactions were largely confined to the binding pocket without inducing major allosteric dynamic shifts captured at this timescale.

Finally, the binding free energies (Δ*G*_bind_) and their constituent energy components were estimated using the molecular mechanics/generalized born surface area (MM/GBSA) method on trajectory snapshots (**Table** **7** and **8**). The calculations predicted significant differences in binding affinity. Guanosine demonstrated the most favorable binding free energy (Δ*G*_bind_ = −27.06 ± 2.91 kcal mol^−1^), followed by 2,6,7‐trimethyldecane (Δ*G*_bind_ = −22.68 ± 2.41 kcal mol^−1^) and 2,2‐dimethoxybutane (Δ*G*_bind_ = −20.03 ± 1.53 kcal mol^−1^). Methyl butyrate exhibited moderate predicted affinity (Δ*G*_bind_ = −10.89 ± 3.93 kcal mol^−1^). In contrast, levoglucosan (Δ*G*_bind_ = +5.2 ± 4.74 kcal mol^−1^) and glycerin (Δ*G*_bind_ = +0.99 ± 0.4 kcal mol^−1^) showed unfavorable positive or near‐zero binding energies, suggesting weak or nonspecific interactions. Strikingly, allyl isothiocyanate yielded a highly unfavorable calculated binding energy (Δ*G*_bind_ = +65.89 ± 13.54 kcal mol^−1^), which is fully consistent with its high RMSD values and observed instability within the binding site.

**Table 7:** Main binding energy components from MMGBSA analysis.

Compounda)	Δ*G*_bind_ [kcal mol^−1^]	Δ*G*_total_ [kcal mol^−1^]	*E*_int_ [kcal mol^−1^]
2,2‐dimethoxybutane	−20.03 ± 1.53	−21.88 ± 1.53	1.85 ± 0.11
Allyl Isothiocyanate	65.89 ± 13.54	−0.12 ± 1.05	66.0 ± 13.5
Levoglucosan	5.2 ± 4.74	−19.26 ± 4.74	24.45 ± 0.11
Methyl butyrate	−10.89 ± 3.93	−16.45 ± 1.5	5.55 ± 3.63
Guanosine	−27.06 ± 2.91	−39.27 ± 2.91	12.2 ± 0.09
Glycerin	0.99 ± 0.4	0.08 ± 0.38	0.91 ± 0.12
2,6,7‐trimethyldecane	−22.68 ± 2.41	−29.35 ± 2.4	6.67 ± 0.12

a) **Note:** All energies are reported in kcal mol^−1^. Δ*G*_bind_: Binding free energy; Δ*G*_total_: Total free energy; *E*_int_: Internal energy.

**Table 8:** Detailed energy components from MMGBSA analysis [kcal mol^−1^].

Compounda)	Δ*E*_vdw_ [kcal mol^−1^]	*E*_elec_ [kcal mol^−1^]	*E*_gb_ [kcal mol^−1^]	*E*_surf_ [kcal mol^−1^]	*E*_ga_ [kcal mol^−1^]	*E*_solv_ [kcal mol^−1^]
2,2‐dimethoxybutane	−21.04 ± 1.77	−12.5 ± 1.54	15.06 ± 1.04	−3.39 ± 0.08	−33.54 ± 1.7	11.67 ± 1.03
Allyl Isothiocyanate	−0.64 ± 1.71	−1.61 ± 24.32	2.24 ± 24.34	−0.1 ± 0.31	−2.25 ± 24.69	2.13 ± 24.26
Levoglucosan	−17.2 ± 2.93	−29.36 ± 10.43	30.52 ± 4.39	−3.22 ± 0.12	−46.56 ± 8.47	27.3 ± 4.33
Methyl butyrate	−16.56 ± 1.72	−8.27 ± 2.74	11.2 ± 1.82	−2.81 ± 0.11	−24.83 ± 2.71	8.39 ± 1.82
Guanosine	−32.6 ± 2.72	−64.11 ± 5.5	62.67 ± 3.25	−5.23 ± 0.15	−96.71 ± 4.99	57.44 ± 3.21
Glycerin	−0.08 ± 0.37	−0.23 ± 1.22	0.39 ± 1.34	−0.01 ± 0.06	−0.31 ± 1.47	0.39 ± 1.31
2,6,7‐trimethyldecane	−32.72 ± 2.44	−0.04 ± 0.16	8.01 ± 0.62	−4.6 ± 0.2	−32.76 ± 2.48	3.41 ± 0.57

a) **Note:** All energies are reported in kcal mol^−1^. Δ*E*_vdw_: van der Waals energy; *E*_elec_: Electrostatic energy; *E*_gb_: Generalized Born energy; *E*_surf_: Surface energy; *E*_ga_: Gas phase free energy; *E*_solv_: Solvation free energy.

Decomposition of the binding energy revealed the driving forces for the interactions. The strong affinity of guanosine was attributed to substantial favorable contributions from both van der Waals (Δ*E*_vdw_ = −32.6 kcal mol^−1^) and electrostatic (*E*_elec_ = −64.11 kcal mol^−1^) interactions, which compensated for the significant unfavorable polar solvation energy (*E*_gb_ = 62.67 kcal mol^−1^). For the hydrophobic ligand 2,6,7‐trimethyldecane, binding was predominantly driven by favorable van der Waals interactions (Δ*E*_vdw_ = −32.72 kcal mol^−1^) and favorable nonpolar solvation contributions (*E*_surf_ = −4.6 kcal mol^−1^), with negligible electrostatic involvement. 2,2‐dimethoxybutane binding involved favorable van der Waals (Δ*E*_vdw_ = −21.04 kcal mol^−1^) and moderate electrostatic (*E*_elec_ = −12.5 kcal mol^−1^) terms. The unfavorable binding of levoglucosan resulted from a large polar desolvation penalty (*E*_gb_ = 30.52 kcal mol^−1^) outweighing its favorable van der Waals and electrostatic terms. The extremely poor calculated affinity for allyl isothiocyanate stemmed from minimal favorable direct interaction terms (Δ*E*_vdw_ and *E*_elec_) coupled with potentially unfavorable internal strain or repulsive interactions averaged over its unstable trajectory, as reflected also in its positive *E*_int_ value. These energetic analyses, combined with the structural stability data, provide a comprehensive view of the differential interactions between the CviR LBD and the tested ligands.

In our study, an in silico molecular docking analysis was performed to investigate the binding potential of the chemical constituents of *A. serpyllifolia* extract to the CviR receptor in detail. The calculated binding energy data revealed that the compounds present in the extract could inhibit the CviR receptor with different binding affinities. Levoglucosan exhibited the highest affinity with a binding energy of −6.8 kcal mol^−1^, while guanosine followed closely with a binding energy of −6.2 kcal mol^−1^. These findings suggest that both compounds can strongly bind to the CviR receptor and may contribute to possible inhibition mechanisms. Allyl isothiocyanate showed a moderate affinity with a binding energy of −5.1 kcal mol^−1^, indicating its potential interaction with the receptor. Other compounds, including 2,2‐dimethoxybutane, glycerin, methyl butyrate, and 2,6,7‐trimethyldecane, exhibited binding energies ranging from −4.1 to −4.7 kcal mol^−1^, indicating lower affinities for the receptor. This suggests that their impact on CviR inhibition may be more limited compared to other identified compounds. Overall, the results indicate that certain compounds in *A. serpyllifolia* extract may target QS mechanisms by binding to the CviR receptor and acting as antagonists. In this context, isolating active compounds from the extract could lead to the development of novel antimicrobial agents with QS inhibition potential. By disrupting bacterial communication systems, QS inhibition can help prevent biofilm formation, virulence factor expression, and antibiotic resistance. Therefore, further biological and pharmacological investigations of *A. serpyllifolia* extract and its chemical constituents could be of great importance for potential therapeutic applications. In a study, it was shown that the components of *F. suspense* could inhibit CviR, particularly with Pinoresinol's binding energy of (−26.02 kcal mol^−1^) and its binding affinity. This result supports the potential of *F. suspense* components to target QS pathways and disrupt bacterial communication and is consistent with the findings of our study.^[^ ^40^ ^]^ Blocking the QS mechanism could be an important approach in treating various infections caused by this organism. Molecular docking analyses confirmed the anti‐QS potential of bioactive compounds from *Cladosporium* spp., with docking scores ranging between –5.2 and –9.5 kcal mol^−1^, supporting their ability to bind to CviR. The inhibition of the QS mechanism could aid in the treatment of various infections caused by *C. violaceum*. Molecular docking results have confirmed the anti‐QS potential of chemical compounds from *Cladosporium* spp. by binding to CviR with docking scores ranging from –5.2 to –9.5 kcal mol^−1^. The binding energies of *A. serpyllifolia* chemical components to the CviR receptor range from –4.1 to –6.8 kcal mol^−1^, which is consistent with the results of the study by Murali et al. (2023).^[^ ^41^ ^]^

## 3 Conclusions

The extract of *A. serpyllifolia* demonstrates significant antioxidant and strong iron chelation activities. This extract also exhibits potent antimicrobial, antibiofilm, and QS activities. Molecular docking analyses revealed that some compounds strongly interact with the CviR receptor, suggesting that these compounds may inhibit the QS system through antagonist effects. Additionally, MD simulations confirmed the stability of the protein–ligand interactions, indicating that some components could serve as potential candidates for drug development.

## 4 Experimental Section

4.1

4.1.1

##### Collection of Plant Material

Plant specimens of *A. serpyllifolia* were collected on 10 May 2023 from the stable dune tops located in Hürriyet District, Çarşamba, Samsun Province, Turkey (Figure S1, Supporting Information). The sampling site was selected based on the presence of homogeneous *A. serpyllifolia* populations within the dominant plant community of *Hippophae rhamnoides*. The identification of the collected specimens was carried out by Dr. Alper Durmaz. The primary reference used for identification was the *Flora of Turkey*,^[^ ^42^ ^]^ and the current taxonomic status and valid scientific name of the species were verified using online taxonomic databases such as Bizim Bitkiler and Plants of the World Online (POWO), along with recent literature.^[^ ^5^, ^6^, ^7^ ^]^ Herbarium specimens belonging to this species were deposited in the Herbarium of the Department of Biology, Faculty of Science, Ondokuz Mayıs University, under the accession number OMUB‐7802.

##### Plant Material Extraction

The above‐ground parts of freshly collected *A. serpyllifolia* were washed with distilled water and cleaned, then dried in an oven at 40 °C for two days. After drying, the above‐ground parts were ground into a fine powder using a blender. The plant samples were preserved by oven drying. From the dried samples, 100 grams were placed into separate containers, and 1 liter of methanol was added to each. These solutions were kept in the dark for 72 h (3 days). After this period, the methanolic extracts were separated by filtration through filter paper. Subsequently, the solvents were evaporated under reduced pressure at 40 °C using a rotary evaporator (Heidolph, Germany) and stored at 4 °C until further use.^[^ ^43^ ^]^

In the compositional analyses performed on *A. serpyllifolia*, the above‐ground parts of the plant were preferred. This choice was primarily due to the absence of a well‐defined storage root structure in this species and the low root‐to‐shoot ratio. Furthermore, in short‐lived, herbaceous species like *A. serpyllifolia,* most biologically active metabolites are concentrated in the above‐ground parts, such as leaves, stems, and flowers. Therefore, the above‐ground parts provide more efficient and representative material for the detection of target phenolic compounds, flavonoids, and other secondary metabolites. Additionally, the weak root structure limits root sampling in terms of both ecological sustainability and the amount of plant material available. For these reasons, the above‐ground parts were selected for this study.

##### 2,2‐diphenyl‐1‐picrylhydrazyl (DPPH) Radical Scavenging Activity

The DPPH test technique was used.^[^ ^37^ ^]^ *A. serpyllifolia* extract was prepared at different concentrations (1, 0.5, 0.25, 0.125, 0.0625, and 0.03125 mg mL^−1^). A DPPH methanol solution was prepared at 80 μg mL^−1^. A 2 mL of the DPPH solution was added to each extract solution, and the mixtures were vortexed. The reaction mixtures were incubated at 27 °C for 30 min. At the end of the incubation period, the absorbance of the reaction mixtures was measured at 517 nm using UV spectrophotometry. Butylated hydroxytoluene (BHT) was used as the standard control. BHT stock solution was prepared at different concentrations (500, 250, 125, 62.5, 32.25, and 16.125 μg mL^−1^). All measurements were performed in triplicate. A calibration curve was prepared. The IC_50_ values of the extracts and ethylenediaminetetraacetic acid (EDTA) were calculated. The antioxidant activity of the *A. serpyllifolia* extract was evaluated using the following formula

(1) $$\text{DPPH scavenging activity }\left(\%\text{ inhibition}\right)=\left[\left(A_{\text{\_control}}-A_{\text{\_sample}}\right)/A_{\text{\_control}}\right]\times 100$$

##### Determination of Ferrous Ion Chelating Capacity

Ferrous ion chelating capacity was determined using a previously reported spectrophotometric method Dinis et al. The extract of *A. serpyllifolia* was prepared in different concentrations (1, 0.5, 0.25, 0.125, 0.0625, and 0.03125 mg mL^−1^). 1.6 mL of the extract, 2.16 mL of distilled water, and 80 μl of 2 mM FeCl_2_ were mixed in a test tube. The reaction was initiated by adding 160 μl of 5 mM ferrozine. The solutions were thoroughly mixed and allowed to stand at room temperature (25 °C) for 10 min. After incubation, the absorbance of the reaction mixtures was measured at 562 nm using UV spectroscopy. EDTA was used as the standard control. The EDTA stock solution was prepared at 500 μg mL^−1^ and serially diluted by half to obtain six different concentrations. The IC_50_ values were calculated, and a calibration curve was constructed. All measurements were performed in triplicate. The ferrous ion chelating ability was calculated using the following formula.^[^ ^44^ ^]^

The antioxidant activity of the *A. serpyllifolia* extract was evaluated using the following formula

(2) $$\text{Ferrous ion chelating capacity }\left(\%\text{ inhibition}\right)=\left[\left(A_{\text{\_control}}-A_{\text{\_sample}}\right)/A_{\text{\_control}}\right]\times 100$$

##### Total Phenolic Content

The Folin–Ciocalteu reagent method was used. Plant extracts were diluted to a concentration of 1 mg mL^−1^. A 0.5 mL was taken from these solutions and mixed with 2.5 mL of Folin–Ciocalteu reagent and 2 mL of 7.5% NaHCO_3_. The Folin–Ciocalteu reagent had been previously diluted tenfold with water. The mixture was incubated at 45 °C for 15 min. After incubation, the absorbance of the reaction mixture was measured at 765 nm using a UV spectrophotometer (Epoch; Bio‐Tek, Winooski, VT, USA). A calibration curve was prepared. Each experiment was performed in triplicate. The results were expressed as GAEs.^[^ ^45^ ^]^

##### Total Flavonoid Content

The total flavonoid content was measured using a previously reported spectrophotometric method by Dewanto et al. The procedure follows: extracts from each plant material (1 mL containing 0.1 mg mL^−1^) were diluted with water (4 mL) in a 10 mL volumetric flask. Initially, 5% NaNO_2_ solution (0.3 mL) was added to each flask. At the 5th minute, 10% AlCl_3_ solution (0.3 mL) was added. At the 6th minute, 1.0 M NaOH (2 mL) was added. Then, water (2.4 mL) was added to the reaction flask and mixed well. The absorbance of the reaction mixture was measured at 510 nm using a UV spectrophotometer (Epoch; Bio‐Tek, Winooski, VT, USA). A calibration curve was prepared. Each experiment was performed in triplicate. The results were expressed as QEs.^[^ ^46^ ^]^

##### Bacterial Strains and Culture Conditions

*Staphylococcus aureus* ATCC 25923, *Bacillus cereus* NRRL B‐3711, *Escherichia coli* ATCC 25922, *Pseudomonas aeruginosa* ATCC 27853, *P. aeruginosa* PA01, and *C. violaceum* ATCC 12472 isolates were grown in sterile Luria Bertani (LB) broth; *Candida albicans* ATCC 10231 strain was cultured in sterile Sabouraud Dextrose Broth.

##### Determination of Minimal Antibacterial Concentration

The antimicrobial activity of *A. serpyllifolia* methanol extract against bacteria and yeast strains was screened by the microdilution method according to recommendations of CLSI reference methods for bacteria with M07‐A7 (CLSI, 2018) and fungi with M27‐A3 (CLSI, 2018). MIC, MBC, and MFC were determined by using 96‐well plates, and the analysis was carried out in triplicate.^[^ ^47^ ^]^ AZT and ketoconazole were positive controls for bacteria and yeast, respectively.

##### Screening for Antibiofilm Activity

To determine the antibiofilm activity of *A. serpyllifolia* extract against biofilm‐forming strains, *P. aeruginosa* ATCC 27853 and *P. aeruginosa* PA01 were used in the microplate‐based crystal violet staining assay modified from the previous studies.^[^ ^48^ ^]^ A 10 μL of overnight bacterial growth (1.5 × 10^8^ cfu) and *A. serpyllifolia* methanol extract were loaded into LB medium (supplemented with 1% w/v glucose) in a dose‐dependent manner (MIC and sub‐MIC doses) with a final volume of 200 μL in a 96‐well microplate and incubated at 37 °C for 24 h. The planktonic cells were removed by washing with sterile water three times, and the cells attached to the wall of the wells were fixed with 200 μL of methanol for 15 min. After emptying the wells and air‐drying, the surface‐adherent biofilm was stained with a 0.1% crystal violet solution. After 15 min, the unbound crystal violet was washed with sterile distilled water. Finally, the adherent biofilm‐bound crystal violet was dissolved with ethanol (95%), and the optical density was measured at a wavelength of 570 nm using an enzyme‐linked immunosorbent assay (ELISA) plate reader. The experiment was carried out in four replications. Bacteria and medium were used as negative controls, and AZT was used as a positive control. The percentage of biofilm inhibition was calculated using the following formula

(3) [(OD growth control −OD sample)/OD growth control]×100

##### Screening for Violacein Inhibition (Anti‐QS Activity)

To screen the violacein inhibition (anti‐QS) activity of *A. serpyllifolia* methanol extract, the quantification of violacein pigment was performed using the method described by Vijayan et al. with slight modifications. *C. violaceum* ATCC 12472 was incubated in LB broth and kept at 30 °C for 24 h. Bacterial turbidity was then adjusted to 0.5 McFarland, equivalent to 1.5 × 108 cfu mL^−1^, using a UV‐visible spectrophotometer. One mL of this bacterial suspension was added to a conical flask containing LB broth and plant extracts to achieve the sub‐MIC concentrations. Bacteria and broth only served as a negative control, while 3 μg mL^−1^ AZT as a positive control. After overnight incubation, 1 mL from each flask was transferred to tubes and centrifuged at 13 000 rpm for 10 min to precipitate violacein. The resulting pellets were resuspended in 1 mL DMSO. After vigorous mixing for 30 s to solubilize the violacein pigment, the samples were centrifuged at 13 000 rpm for 10 min to remove the cells. Two hundred microlitres of the suspension were transferred to a 96‐well microtitre plate and the absorbance was measured spectrophotometrically at 585 nm (BioTek Epoch plate spectrophotometer). The experiments were carried out in three independent trials in triplicates. The mean absorbance of the samples was determined and the violacein inhibition was calculated using the equation below

(4) % Violacein Inhibition=OD (control)−OD (test)*100 OD (control)

where OD (control) is the average OD of the negative control. OD (test) is the average OD of the test for each concentration of the extract.^[^ ^49^ ^]^

##### GC‐MS Analysis

To determine the chemical constituents of the optimized materials, gas chromatography coupled with mass spectrometry was carried out, drawing methodological reference from the previous studies.^[^ ^37^ ^]^ Following centrifugation of the samples at 3500 revolutions per minute for 10 min, the clarified upper phase was separated for analysis. Measurements were performed using a SHIMADZU GCMS‐QP2010 system paired with an AOC‐5000 automatic sampler. An Rxi‐5MS capillary column (dimensions: 30 m × 0.25 mm × 0.25 μm) was utilized for compound separation. The detector was set to monitor ions in the 30–450 Dalton range. Methanolic extracts of plant material, prepared via liquid phase extraction, were diluted 1:100 v/v and introduced into 1.5 mL vials. Identification of molecular structures was accomplished by referencing the NIST spectral library.

##### Molecular Docking Procedure: Ligand Preparation

Phytochemical compounds identified through GC‐MS analysis were selected for docking studies, and their corresponding structures were retrieved from the PubChem database. The molecular files, initially in SDF format, were converted into PDB format using Discovery Studio Visualizer. Torsional flexibility was incorporated by adjusting rotatable bonds to better represent dynamic ligand conformations. Subsequently, PyRx software, incorporating the AutoDock Vina backend, was employed to convert the structures into PDBQT format suitable for docking protocols.

##### Receptor Preparation

The 3D structure of the CviR LBD complexed with its native ligand (PDB ID: 3QP1) was downloaded from the RCSB Protein Data Bank (https://www.rcsb.org/). To make the protein suitable for molecular docking, preparatory steps were applied, these included the elimination of crystallographic water molecules, correction of atomic inconsistencies, and assignment of partial charges. The receptor was then exported in PDBQT format using AutoDock Tools from the AutoDock 4.2 suite.

##### Docking Simulations and Binding Energy Evaluation

Ligand–receptor interaction studies were carried out using AutoDock‐based platforms, where both protein and ligand files were processed through dedicated modules, ensuring accurate configuration.^[^ ^50^ ^]^ Docking simulations aimed to identify energetically favorable binding modes, with output poses ranked by binding affinity. The structure showing the lowest binding energy was chosen for interaction analysis. Postdocking visualization and confirmation of molecular interactions were conducted using Discovery Studio Visualizer, ensuring both structural compatibility and potential biological relevance.

In our investigation, GROMACS 2024.4 software was employed for executing all MD simulations.^[^ ^51^ ^]^ Parameter determination for nonconventional molecular structures was performed through the acpype toolkit,^[^ ^52^ ^]^ which utilizes AM1‐BCC methodology to accurately calculate partial charge distributions across ligand architectures.^[^ ^53^ ^]^ For protein components, characterization was conducted within the Amber99SB‐ILDN force field framework,^[^ ^54^ ^]^ while aqueous environments were represented using the TIP3P water model configuration.^[^ ^55^ ^]^

System preparation involved energy minimization procedures employing the steepest descent algorithm until force magnitudes diminished below the 1000.0 kJ mol^−1^ threshold. Computational boundaries implemented periodic conditions with 1.0 nm truncation distances applied to both electrostatic and van der Waals interaction calculations.

Our equilibration protocol incorporated sequential thermodynamic ensemble approaches. The initial constant number of particles, volume, and temperature (NVT) equilibration extended through 300 ps, during which positional restraints were maintained on both protein and ligand structures. This phase operated under Berendsen thermostat regulation at 310 K,^[^ ^56^ ^]^ utilizing 2 fs time increments with leap‐frog integration methodology. Subsequently, constant number of particles, pressure, and temperature (NPT) equilibration proceeded for 1 ns, implementing velocity‐rescale temperature regulation^[^ ^57^ ^]^ in conjunction with Berendsen pressure management to maintain isotropic 1.0 bar conditions with a 2 ps coupling parameter.

Production simulations were executed within the NPT ensemble framework utilizing Parrinello–Rahman pressure regulation mechanisms.^[^ ^58^ ^]^ These extended analyses continued through 100 ns, representing 50 million computational iterations at 2 fs intervals. Hydrogen‐containing bond distances were constrained via the LINCS algorithm (fourth‐order approximation with single iteration constraints).^[^ ^59^ ^]^ For calculating long‐range electrostatic interactions, particle mesh Ewald methodology was implemented with 1.2 nm truncation and 0.16 nm Fourier spacing parameters.^[^ ^60^ ^]^ Nonbonded interaction computations utilized the Verlet scheme with grid‐based neighbor identification protocols.

Binding energy quantification was achieved through gmx_MMPBSA (which utilizes the molecular mechanics poisson–Boltzmann surface area (MMPBSA) approach) analytical tools.^[^ ^61^ ^]^ These assessments employed MMGBSA approaches on trajectory frames extracted from later simulation periods, with interaction entropy approximations determined across segmented intervals (50‐frame segments) at physiological temperature conditions (310 K).

##### Statistical Analysis

All data were expressed as means ± standard deviation (S.D.). Standard deviation values were calculated using SPSS Statistics version 21.0

## Conflict of Interest

The authors declare no conflict of interest.

## Supporting information

Supplementary Material

## Data Availability

The data that support the findings of this study are available from the corresponding author upon reasonable request.
