# Supplementary material for: Arenaria serpyllifolia as a Natural Antiviolaceum Agent: Phytochemical, Biological, and Molecular Approaches
Source: ChemistryOpen. 2025 Jul 15;14(11):e202500236. doi: 10.1002/open.202500236 (PMC12598791; doi:10.1002/open.202500236)
Supplement: Supplementary file 1 — Supplementary Material [file OPEN-14-e202500236-s001.pdf]

Arenaria serpyllifolia as a Natural Anti-violaceum Agent: Phytochemical, Biological, and Molecular Approaches

Meryem Burcu Külahcı[1], Betül Aydın[1], Emine Incilay Torunoğlu[2], Zekeriya Düzgün[3], Alper Durmaz[4], Erdi Can Aytar[5*]

[1]Gazi Üniversiteyi, Faculty of Science, Department of Biology, 06500, Ankara, Türkiye

[2]Necmettin Erbakan University, Faculty of Medicine, Department of Medical Biochemistry, 42090, Konya, Türkiye

[3]Giresun University, Faculty of Medicine, Department of Medical Biology, Giresun, Türkiye

[4]Artvin Çoruh University, Ali Nihat Gökyiğit Botanical Garden Application and Research Center, 08000, Artvin, Türkiye

[5]Usak University Faculty of Agriculture Department of Horticulture, 64200, Uşak, Türkiye

Correspondence: Erdi Can Aytar e- mail: erdicanaytar@gmail.com

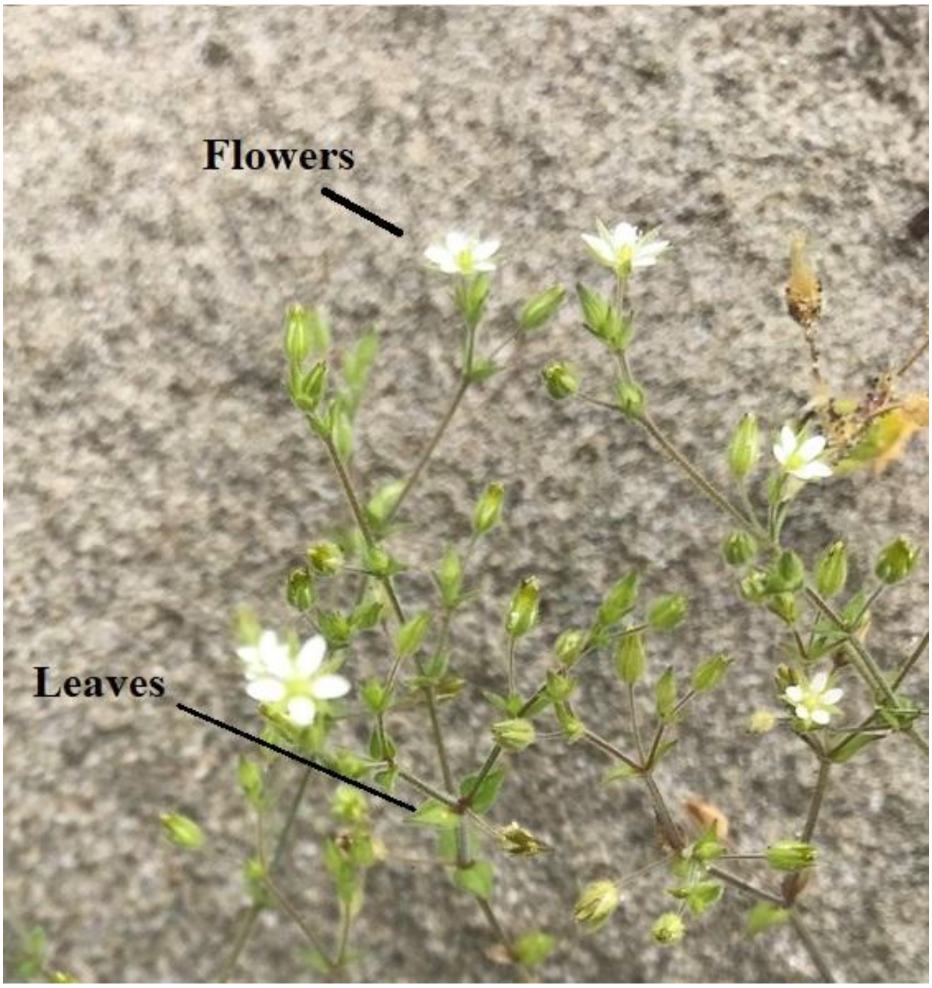

Figure SI 1. The morphological appearance of the above-ground parts of *A. serpyllifolia* plant
